# Association of pregnancy history and unhealthy lifestyle with biological age acceleration: a large cross-sectional study

**DOI:** 10.3389/fpubh.2026.1761874

**Published:** 2026-03-17

**Authors:** Yuting Yao, Shaohua Yin, Keyi Si, Dan Li, Lei Yuan, Yingying Yang, Zhen Li, Guizhu Wu

**Affiliations:** 1 Shanghai Key Laboratory of Maternal Fetal Medicine, Department of Gynecology, School of Medicine, Shanghai First Maternity and Infant Hospital, Shanghai Institute of Maternal-Fetal Medicine and Gynecologic Oncology, Tongji University, Shanghai, China; 2 Department of Medical Engineering, Peking University Third Hospital, Beijing, China; 3 Department of Environmental Health, School of Public Health, Shanghai Jiao Tong University School of Medicine, Shanghai, China; 4 Beijing Key Laboratory of Cardiovascular Receptors Research, State Key Laboratory of Vascular Homeostasis and Remodeling, NHC Key Laboratory of Cardiovascular Molecular Biology and Regulatory Peptides, Department of Cardiology and Institute of Vascular Medicine, Peking University, Beijing, China; 5 Department of Health Management, Naval Medical University, Shanghai, China; 6 Key Laboratory of Maternal Fetal Medicine, Clinical Research Unit, School of Medicine, Shanghai First Maternity and Infant Hospital, Shanghai Institute of Maternal-Fetal Medicine and Gynecologic Oncology, Tongji University, Shanghai, China

**Keywords:** biological aging, Klemera-Doubal Method, PhenoAge biological age, pregnancy history, unhealthy lifestyle

## Abstract

**Objective:**

This study aimed to investigate the association of pregnancy history with biological age (BA) and BA acceleration, and to explore joint effect with lifestyle factors.

**Methods:**

This cross-sectional study used data from the UK Biobank, a population-based cohort recruited across UK between 2006 and 2010, with followed up until the end of 2022. Female participants with available data on chronological age, pregnancy history, lifestyle factors, and clinical biochemistry biomarkers were included. BA and aging acceleration were estimated using Klemera-Doubal Method (KDM BA) and PhenoAge algorithms. The KDM BA, PhenoAge, KDM BA acceleration, and PhenoAge acceleration were calculated. Multivariable general linear regression models were used to assess the associations of pregnancy history alone and combined with lifestyle with BA and BA acceleration.

**Results:**

Among 137,218 participants [mean (SD) age, 55.73 (8.01) years], 115 675 (84.3%) reported a history of pregnancy. Compared to those who had never been pregnant, women with a history of pregnancy had a decrease in BA [β = −0.312 (95% CI, −0.432 to −0.191) for KDM BA; β = −0.242 (95% CI, −0.302 to −0.183) for PhenoAge], and BA acceleration [β = −0.021 (95% CI, −0.034 to −0.009) for KDM BA acceleration; β = −0.088 (95% CI, −0.102 to −0.075) for PhenoAge acceleration]. Women with unhealthier lifestyle behaviors had higher BA and BA acceleration, regardless of pregnancy history (*P* for interaction > 0.05).

**Conclusions:**

In this large population-based study, pregnancy was associated with lower BA and BA acceleration, while an unhealthier lifestyle was associated with higher BA and BA acceleration. These findings suggest that both reproductive and lifestyle factors may play a role in developing anti-aging interventions and guiding healthy aging policies.

## Introduction

Between 2020 and 2050, the global population aged 80 years and older is projected to more than triple, reaching approximately 426 million (1). As population's age and life expectancy rises, age-related diseases impose an increasing burden on healthcare systems and quality of life (2). Understanding the factors associated with aging is essential for developing strategies to promote healthy aging and reduce the societal and economic costs of age-related health deterioration. While chronological aging is commonly used as an indicator of aging, it does not fully capture individual differences in biological aging, as aging rates vary significantly even among individuals of the same chronological age (3, 4). To address this limitation, biological age acceleration, an emerging biomarker-based aging measure derived from clinical indicators, provides a comprehensive approach to assessing geroprotective interventions, identifying risk factors, and elucidating underlying mechanisms underlying aging (4).

Pregnancy induces significant physiologic changes, leading to metabolic, cardiovascular, and immunological adaptations (5, 6). Most studies examining the association between reproductive history and biological aging acceleration have focus on DNA methylation-based aging measures (7–9). However, evidence regarding its association with biological age acceleration estimated using Klemera–Doubal Method biological age (KDM BA) or phenotypic age (PhenoAge), derived from clinical biochemistry biomarkers (10), remained limited (11–13), and inconsistent (7, 13, 14). A cohort study of 54 pregnant Swedish women reported a progressive increase in PhenoAge acceleration from prepregnancy to 2–4 days postpartum (11, 12). In contrast, a recent cross-sectional study of young Filipino women (aged 20–22 years) found a non-significant negative relationship between gravidity and PhenoAge acceleration (7). Similarly, a cross-sectional study of American women reported that parity was not associated with KDM-BA acceleration among premenopausal women but was observed in postmenopausal women (14).

Meanwhile, unhealthy lifestyle behaviors, such as smoking, excessive alcohol consumption, and poor diet, are well-established contributors to accelerated aging (15–17). Notably, pregnancy is often associated with changes in lifestyle patterns, which may influence aging trajectories. Understanding the independent and combined effects of pregnancy and lifestyle factors on biological aging is crucial for developing targeted public health interventions.

Using data from the UK Biobank, a large population-based cohort study of approximately 500,000 participants aged 39 to 71 years, we investigated the association of pregnancy with biological age and its acceleration. In addition, we examined the interaction between pregnancy and unhealthy lifestyle behaviors on these aging measures.

## Material and methods

### Data source and participants

The study used data from the UK Biobank (UKB), a large-scale, population-based prospective cohort that recruited over 500,000 participants aged 39 to 71 years from 22 assessment centers across England, Scotland, and Wales between 2006 and 2010. At baseline, UKB collected extensive sociodemographic, lifestyle, and health-related data through self-administered questionnaires, in-person interviews, physical measurements, and biological sample collection. Additionally, UKB linked participants' health records to national databases, including hospital admissions and mortality data, enabling long-term follow-up. Further details on the UKB study design and population have been described elsewhere (18).

**Figure 1:**
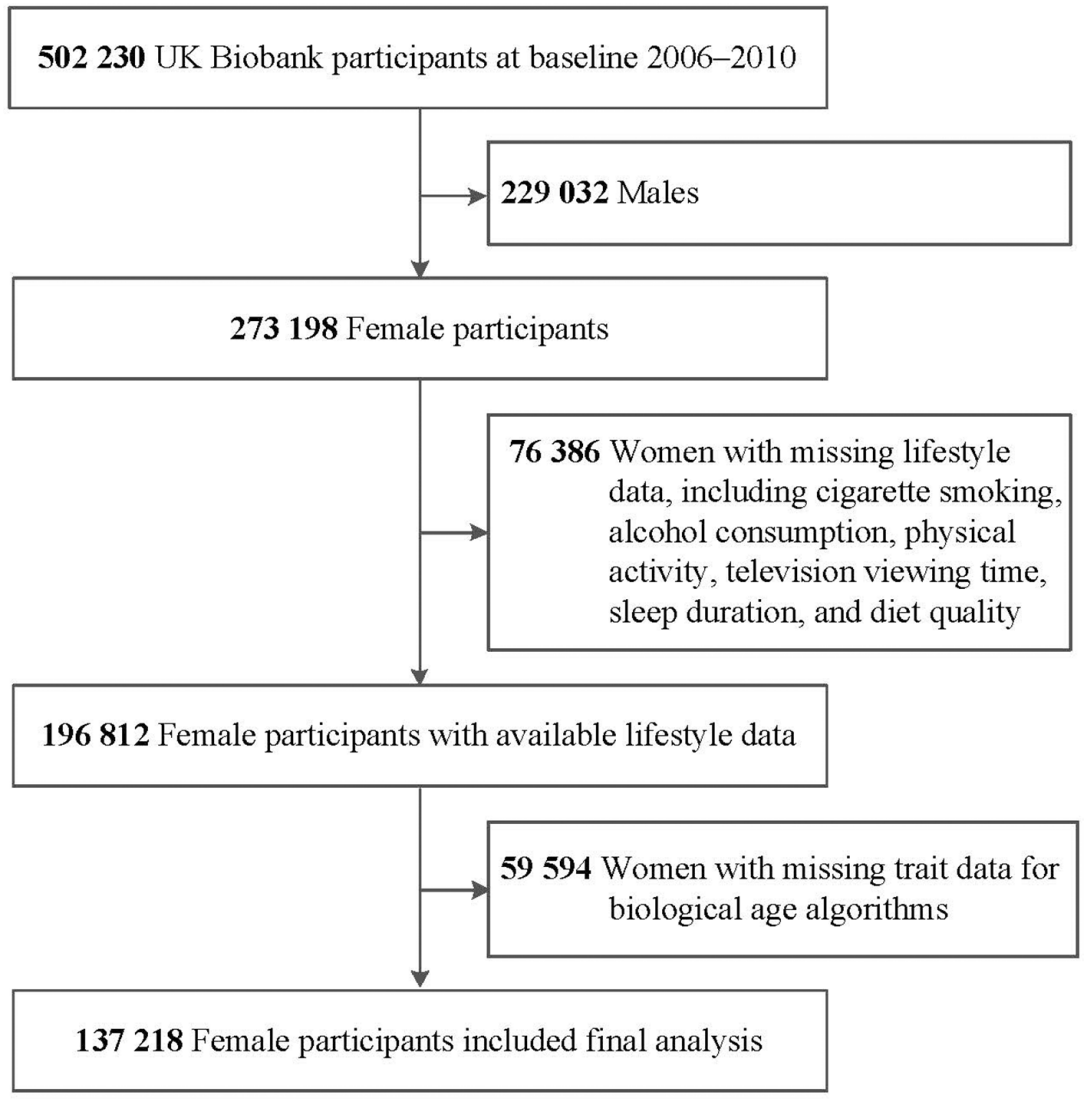
Study flow diagram.

From the initial UKB cohort of 502,230 participants, a total of 273,198 female participants were included in this study. We sequentially excluded 487 individuals with missing information on pregnancy history, 75,899 individuals with missing information on lifestyle factors that were used to generate an unhealthy lifestyle score, and 59,594 individuals without available clinical biomarkers for biological age estimation, finally 137,218 individuals were included in the study (Figure 1).

### Assessment of pregnancy history

Pregnancy history was assessed using self-reported reproductive data collected through questionnaires administered at baseline. Participants provided information on age at first and last live birth, history and number of spontaneous or induced abortions, and stillbirths. Additional details on gestational complications were recorded, including gestational diabetes mellitus, gestational hypertension, eclampsia, hydatidiform mole, gestational edema and proteinuria, ectopic pregnancy, venous complications in pregnancy, hemorrhage in early pregnancy, malnutrition during pregnancy. Participants were categorized as either never pregnant or ever pregnant according to their reproductive information.

### Assessment of unhealthy lifestyle score

Given the interrelated nature of multiple lifestyle factors and their association with accelerated aging (15–17), an unhealthy lifestyle score was generated based on cigarette smoking, alcohol consumption, physical activity, television viewing/sedentary time, sleep duration, fruit and vegetable consumption, oily fish consumption, and red and processed meat consumption, according to a previous Australian study (19). At baseline, participants completed a self-administered touchscreen questionnaire on lifestyle behaviors. Each unhealthy behavior was considered as “at risk” and assigned 1 point: current smoking, daily or almost-daily alcohol consumption, insufficient physical activity (< 150 min/week of moderate-intensity or < 75 min/week of vigorous-intensity exercise), excessive television viewing/sedentary time (≥4 hours/day), suboptimal or excessive sleep duration (< 7 or >9 hours/day), low fruit and vegetable intake (< 400 g/day), insufficient oily fish intake (< 1 portion/week), excessive red meat intake (>3 portions/week), and high processed meat intake (>1 portion/week). The final unweighted unhealthy lifestyle score ranged from 0 (healthiest) to 9 (unhealthiest). For analysis, participants were categorized into three groups: most healthy (score 0–2), moderately healthy (score 3–5), and least healthy (score 6–9). Detailed definitions of the unhealthy lifestyle score and its categories are provided in Supplementary Table S1.

### Construction of biological age and age acceleration

Biological age (BA) was estimated using two widely validated algorithms, the Klemera-Doubal Method (KDM) and PhenoAge, which incorporate clinical biomarkers and physical measurements to predict age-related health outcomes (20, 21). Briefly, KDM BA was derived through forced expiratory volume in 1 s, systolic blood pressure, and seven clinical biomarkers: total cholesterol, glycated hemoglobin, blood urea nitrogen, albumin, creatinine, C-reactive protein, and alkaline phosphatase. PhenoAge was calculated using nine clinical biomarkers: albumin, alkaline phosphatase, creatinine, C-reactive protein, glucose, mean cell volume, red blood cell distribution width, white blood cell count, and lymphocyte ratio. The distribution of these biomarkers was provided in Supplementary Table S2, and BA values were computed using the R package “BioAge” (https://github.com/dayoonkwon/BioAge).

KDM BA estimates an individual's physiological age relative to their chronological age using a biomarker-based regression approach applied to a reference population. The equation for calculating KDM BA incorporates multiple regression lines, each representing the relationship between a specific biomarker and age. The formula is:


$$\begin{array}{l} \text{KDM BA=}\frac{\sum_{i=1}^{\text{m}}\left(x_{i}-q_{i}\right)\left(\frac{k_{i}}{s_{i}^{2}}\right)+\frac{\text{Choronological age}}{s_{\text{BA}}^{2}}}{\sum_{i=1}^{\text{m}}\left(\frac{k_{i}}{s_{i}^{2}}\right)+\frac{1}{s_{\text{BA}}^{2}}} \end{array}$$

where x is the value of biomarker i for the individual, and k_i_, q_i_, and s_i_ are the estimated parameters from the regression model for each biomarker, including the intercept, slope, and the root mean squared error, respectively. The s_BA_ is a scaling factor accounting for the variance in chronological age explained by the biomarker set, determined from the reference population.

The PhenoAge algorithm, developed using elastic-net Gompertz regression, predict biological age based on multivariate analysis of mortality risk using a set of biomarkers. Originally derived from the NHANES III dataset based on 42 biomarkers from, its formula is:


$$\begin{array}{r} \text{PhenoAge=141.50225+}\frac{\ln\;\left(-0.00553\right)\times \ln\;\left(1-\text{Mortality risk}\right)}{0.09165}\\ \text{Mortality risk=1}-{\text{e}}^{-{\text{e}}^{\text{xb}}\left[\exp\left(120*\gamma \right)-1\right]/\gamma } \end{array}$$

where *xb* represents the linear combination of biomarkers from the regression model, and γ is an ancillary parameter estimated from the data.

To evaluate individual variations in BA, we regressed BA against chronological age at biomarker measurement and used the residuals as the indicator of biological aging, referred to as BA acceleration. To facilitate comparison between biological aging measures, BA acceleration was standardized to a mean of 0 and a standard deviation (SD) of 1.

### Covariates

Chronological age was calculated using participants' birthdates and the date of their baseline assessment. Race and ethnicity was self-reported as white, mixed, Asian or Asian British, black or black British, Chinese, or other ethnic group; for analysis, due to small sample sizes in non-White ethnic subgroups, participants were classified into White and non-White groups. Educational level was determined based on the highest self-reported qualification and categorized as high (college or university degree), intermediate (A/AS levels, O-levels, General Certificate of Secondary Education, or equivalent, corresponding to grades 6–12 in the US school system), and low (no formal qualifications) (22). Socioeconomic status was assessed using the Townsend Deprivation Index (TDI), derived from national census data on car ownership, household overcrowding, home ownership, and unemployment aggregated at the postcode level (23). Higher TDI scores indicate greater socioeconomic deprivation. The Charlson Comorbidity Index (CCI) was calculated using ICD-9 and ICD-10 diagnosis codes extracted from hospital administrative data to quantify comorbidity burden. The CCI scores were derived following the original method described by Charlson et al. (24), with weighted scores reflecting comorbidity severity. Body mass index (BMI) was calculated as weight in kilogram divided by squared of height in meter, measured by trained staffs at baseline. BMI was classified according to WHO criteria as underweight (< 18.5 kg/m^2^), normal weight (18.5–24.9 kg/m^2^), overweight (25.0–29.9 kg/m^2^), and obese (≥30.0 kg/m^2^).

### Statistical analysis

Descriptive statistics were used to summarize baseline characteristics, with continuous variables presented as means ± standard deviations (SD) and categorical variables as frequencies (percentages).

General linear regression models were used to examine the associations between pregnancy history and both BA and BA acceleration, and regression coefficients with their corresponding 95% confidence intervals (95% CI) were reported from models. For joint effect analyses, participants were classified into six groups based on pregnancy history and unhealthy lifestyle score categories, with participants who adhered to the healthiest lifestyle and had never been pregnant serving as the reference group. Interaction between pregnancy history and unhealthy lifestyle score was assessed exploratory analyses using likelihood ratio tests, by comparing models with and without cross-product terms. The independent associations of individual lifestyle factors (categorized as at risk vs. not at risk) with BA and BA acceleration was also assessed. A sequence of regression models was performed: (1) adjusted for socioeconomic variables, including chronological age, race and ethnicity, educational level, BMI, and TDI; (2) further adjusted for CCI; (3) further adjusted for unhealthy lifestyle score. Missing data for covariates were not imputed but coded as “unknown,” which was classified as a separate category in regression models.

Several sensitivity analyses were performed to assess the robustness of the findings. First, stratified analyses were conducted by chronological age (39–59 and 60–71 years). Second, the analyses were repeated without adjusting for chronological age to evaluate its potential effect. Third, the primary analysis was repeated using a complete-case sample of 136 058 participants. Fourth, three reproductive factors reflecting pregnancy history—including the number of live births, stillbirths, and miscarriages, were individually included in the models to examine their specific associations with biological age acceleration. Fifth, baseline characteristics were compared between participants excluded due to missing clinical biomarkers data and the total population. Sixth, Finally, additional adjustments were made for gestational diabetes mellitus, hypertensive disorders of pregnancy, and hormone replacement therapy use.

All statistical analyses were performed using SAS 9.4 (SAS Institute, Cary, NC, USA) and R 4.3.1 (R Project for Statistical Computing). A two-tailed *P* < 0.05 was considered statistically significant.

## Results

### Baseline characteristics

Of 137,218 female participants, the mean chronological age at baseline was 55.73 ± 8.01 years, and 115,675 reported a history of at least one pregnancy (Table 1). Compared to women who had never been pregnant, those with a history of pregnancy had a lower TDI (−1.61 ± 2.89 vs. −0.98 ± 3.07) and unhealthy lifestyle score (2.03 ± 1.33 vs. 2.04 ± 1.33), had a higher CCI (0.86 ± 1.54 vs. 0.79 ± 1.50), and prevalence of diabetes (6.1% vs. 5.4%), hypertension (9.6% vs. 7.4%), and overweight and obese (58.3% vs. 53.8%). In addition, women with a history of pregnancy were more likely to be current smokers (8.3% vs. 7.8%), but less likely to engage in excessive alcohol consumption at baseline (17.0% vs. 19.0%) (Table 1).

**Table 1:** Demographic and clinical characteristics of study participants.

**Characteristic**	**Participants**		
	**All (*****N*** = **137,218)**	**Never pregnancy (*****n*** = **21,543)**	**Ever pregnancy (*****n*** = **115,675)**
**Chronological age**	55.73 ± 8.01	53.65 ± 8.02	56.11 ± 7.95
≤ 54	58,550 (42.7)	11,620 (53.9)	46,930 (40.6)
55–59	25,482 (18.6)	3,817 (17.7)	21,665 (18.7)
60–64	31,947 (23.3)	3,823 (17.7)	28,124 (24.3)
≥65	21,239 (15.5)	2,283 (10.6)	18,956 (16.4)
Race and ethnicity			
White	131,175 (95.6)	20,671 (96.0)	110,504 (95.5)
Non-white^a^	5,784 (4.2)	816 (3.8)	4,968 (4.3)
Unknown	56 (0.3)	203 (0.2)	259 (0.2)
Educational level^b^			
High	5,983 (27.8)	28,806 (24.9)	34,789 (25.4)
Intermediate	14,009 (65)	70,267 (60.7)	84,276 (61.4)
Low	1,464 (6.8)	15,970 (13.8)	17,434 (12.7)
Unknown	87 (0.4)	632 (0.5)	719 (0.5)
Townsend deprivation index^c^	−1.51 ± 2.93	−0.98 ± 3.07	−1.61 ± 2.89
BMI at recruitment (kg/m^2^)			
< 18.5	1,017 (0.7)	274 (1.3)	743 (0.6)
18.5–24.9	56,996 (41.5)	9,657 (44.8)	47,339 (40.9)
25–29.9	50,265 (36.6)	7,078 (32.9)	43,187 (37.3)
≥30	28,747 (20.9)	4,499 (20.9)	24,248 (21.0)
Unknown	193 (0.1)	35 (0.2)	158 (0.1)
Unhealthy lifestyle score	2.04 ± 1.33	2.04 ± 1.33	2.03 ± 1.33
Most healthy (0–2)	91,659 (66.8)	14,293 (66.3)	77,366 (66.9)
Moderately healthy (3–5)	44,058 (32.1)	7,014 (32.6)	37,044 (32.0)
Least healthy (6–9)	1,501 (1.1)	236 (1.1)	1,265 (1.1)
Cigarette smoking			
Never/Previous	125,976 (91.8)	19,858 (92.2)	106,118 (91.7)
Current	11,242 (8.2)	1,685 (7.8)	9,557 (8.3)
Alcohol consumption			
≤ 4 times week	113,425 (82.7)	17,442 (81.0)	95,983 (83.0)
Daily or almost daily	23,793 (17.3)	4,101 (19.0)	19,692 (17.0)
Charlson comorbidity index^d^	0.85 ± 1.54	0.79 ± 1.50	0.86 ± 1.54
Diabetes			
No	128,957 (82.7)	20,376 (94.6)	108,581 (93.9)
Yes	8,261 (17.3)	1,167 (5.4)	7,094 (6.1)
Hypertension			
No	124,477 (90.7)	19,945 (92.6)	104,532 (90.4)
Yes	12,741 (9.3)	1,598 (7.4)	11,143 (9.6)
Cancer			
No	117,562 (85.7)	18,386 (85.3)	99,176 (85.7)
Yes	19,656 (14.3)	3,157 (14.7)	16,499 (14.3)

^a^Other includes any races or ethnicities not specifically categorized. ^b^Educational attainment was categorized into three levels: high, defined as holding a college or university degree; intermediate, which includes advanced (A/AS) levels or equivalent, ordinary (O) level, general certificate of secondary education, or equivalent qualifications—corresponding to grades 6–12 in the US education system, where O-level is equivalent to middle school (grades 6–8), and A/AS-level corresponds to high school (grades 9–12); and low, referring to individuals without any of the aforementioned qualifications. ^c^The Townsend Deprivation Index is a measure of socioeconomic status, where 0 represents the mean value for an area, positive values indicate lower socioeconomic status, and negative values indicate higher socioeconomic status. ^d^Body mass index was calculated as body weight in kilogram divided by squared of height in meter.

### Associations of pregnancy history with BA and BA acceleration

In crude analysis, the mean KDM BA, PhenoAge, KDM BA acceleration, and PhenoAge acceleration were 53.64 ± 11.86 years, 48.63 ± 8.75 years, 0.09 ± 0.89 years, and −0.19 ± 0.99 years, respectively. Compared to women who had never been pregnant, those with a history of pregnancy had a higher mean KDM BA (54.01 ± 11.82 vs. 51.66 ± 11.91 years) and PhenoAge (48.96 ± 8.71 vs. 46.85 ± 8.76 years). However, lower values were observed for KDM BA acceleration (0.09 ± 0.89 vs. 0.09 ± 0.9 years) and PhenoAge acceleration (−0.20±0.98 vs. −0.11 ± 1.00 years) (Figure 1).

After adjusting for potential confounders as in model 1, women with a history of pregnancy had a decrease in BA [β = −0.317 (95% CI, −0.438 to −0.196) for KDM BA; β = −0.249 (95% CI, −0.309 to −0.189) for PhenoAge], and BA acceleration [β = −0.019 (95% CI, −0.031 to −0.006) for KDM BA acceleration; β = −0.085 (95% CI, −0.099 to −0.071) for PhenoAge acceleration] (Table 2). These associations remained largely unchanged after further adjustment for CCI and unhealthy lifestyle score in models 2 and 3 [β = −0.312 (95% CI, −0.432 to −0.191) for KDM BA; β = −0.242 (95% CI, −0.302 to −0.183) for PhenoAge], and BA acceleration [β = −0.021 (95% CI, −0.034 to −0.009) for KDM BA acceleration; β = −0.088 (95% CI, −0.102 to −0.075) for PhenoAge acceleration].

**Table 2:** Associations of pregnancy history with biological age and biological age acceleration.

**Model**	**KDM BA**	**PhenoAge**	**KDM BA acceleration**	**PhenoAge acceleration**
Model 1^a^				
Never pregnancy	Reference	Reference	Reference	Reference
Ever pregnancy	−0.317 (−0.438, −0.196)	−0.249 (−0.309, −0.189)	−0.019 (−0.031, −0.006)	−0.085 (−0.099, −0.071)
Model 2^b^				
Never pregnancy	Reference	Reference	Reference	Reference
Ever pregnancy	−0.307 (−0.428, −0.186)	−0.239 (−0.299, −0.180)	−0.021 (−0.034, −0.009)	−0.089 (−0.103, −0.075)
Model 3^c^				
Never pregnancy	Reference	Reference	Reference	Reference
Ever pregnancy	−0.312 (−0.432, −0.191)	−0.242 (−0.302, −0.183)	−0.021 (−0.034, −0.009)	−0.088 (−0.102, −0.075)

Data are regression coefficients with their corresponding 95% confidence intervals.

^a^Model 1 was adjusted for chronological age, race and ethnicity, educational level, BMI, and Townsend deprivation index.

^b^Model 2 was further adjusted for Charlson Comorbidity Index based on model 1.

^c^Model 3 was further adjusted for unhealthy lifestyle score based on model 2.

**Figure 2:**
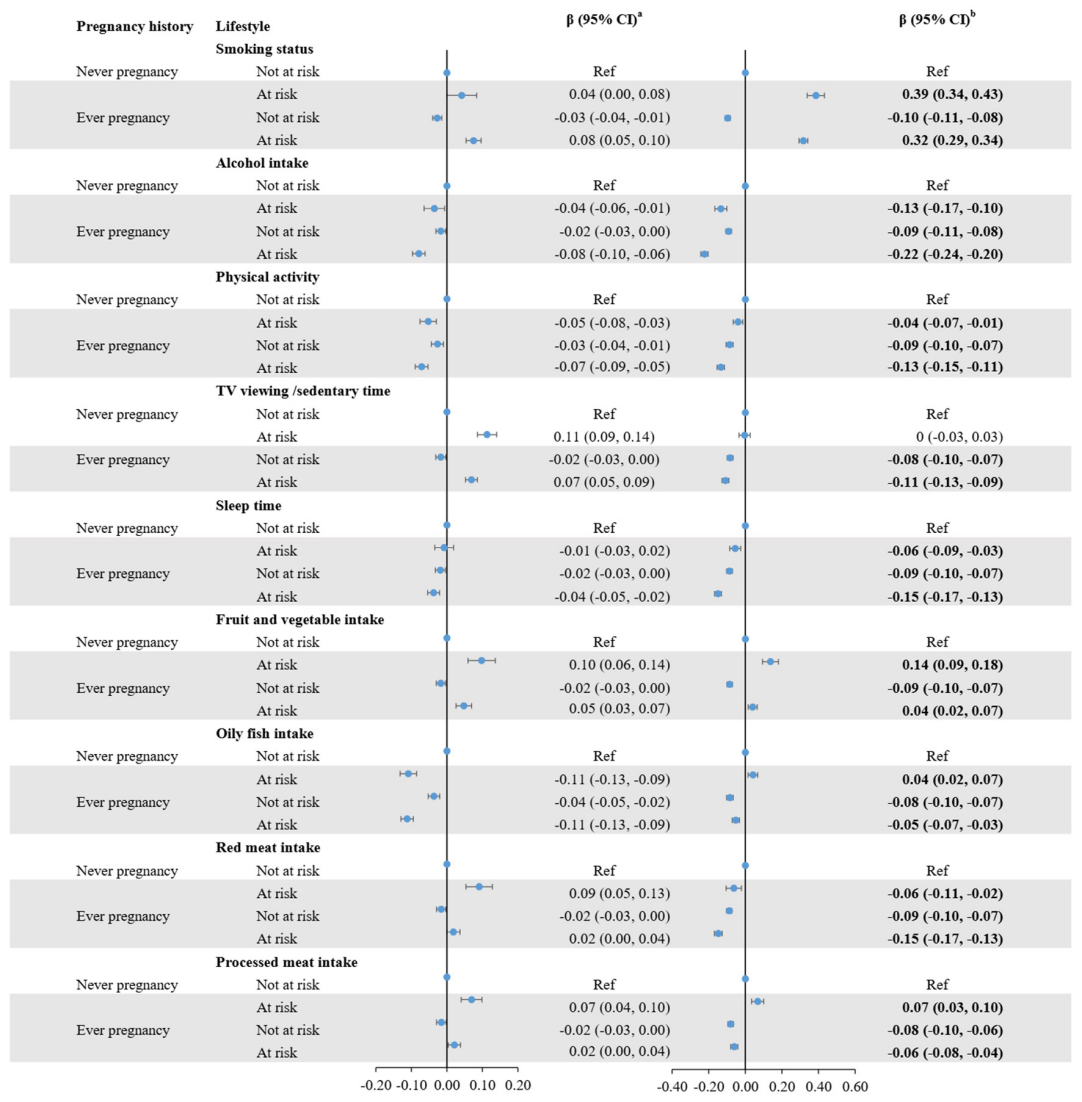
Joint effects of pregnancy history and unhealthy lifestyle in biological age and biological age acceleration. The model was adjusted for chronological age, race and ethnicity, educational level, BMI, Townsend deprivation index, and Charlson Comorbidity Index. ^a^Regression coefficients and 95% confidence intervals for biological age, and ^b^regression coefficients and 95% confidence intervals for biological age acceleration.

In the fully adjusted model examining the joint effect, compared to women who had never been pregnant and with the most healthy lifestyle, those who had never been pregnant and with the least healthy lifestyle had the highest BA [β = 3.396 (95% CI, 2.341 to 4.452) for KDM BA; β = 2.004 (95% CI, 1.772 to 2.237) for PhenoAge] and BA acceleration [β = 0.340 (95% CI, 0.232 to 0.449) for KDM BA acceleration; β = 0.467 (95% CI, 0.412 to 0.522) for PhenoAge acceleration] (Table 3). However, no significant interaction was found between pregnancy history and unhealthy lifestyle score on BA acceleration (*P*_interaction_ > 0.05). For example, in the joint analysis of pregnancy history and smoking status, women who had never been pregnant but were classified as “at risk” smokers showed a higher BA acceleration compared with the reference group of women who had never been pregnant and were not at risk (β = 0.39, 95% CI: 0.34 to 0.43). Similarly, among women with a history of pregnancy, those with at-risk smoking status also showed increased BA acceleration (β = 0.32, 95% CI: 0.29 to 0.34), whereas women with a history of pregnancy but not at-risk smoking status had a lower BA acceleration (β = −0.10, 95% CI: −0.11 to −0.08) (Figure 2). Additionally, analyses adjusting for individual lifestyle factors showed that women classified as “at risk” due to smoking, prolonged TV viewing/sedentary time, inadequate/excessive sleep, insufficient fruit and vegetable intake, and had excessive processed meat intake an increase in BA and BA acceleration compared to those classified as “not at risk” (Supplementary Table S3).

**Table 3:** Joint effects of pregnancy history and lifestyle score with biological age and biological age acceleration.

**Model**	**KDM BA**	**PhenoAge**	**KDM BA acceleration**	**PhenoAge acceleration**
Model 1^a^				
Never pregnancy + most healthy	Reference	Reference	Reference	Reference
Never pregnancy + moderately healthy	1.012 (0.777, 1.248)	0.724 (0.608, 0.840)	0.104 (0.079, 0.128)	0.170 (0.143, 0.197)
Never pregnancy + least healthy	3.581 (2.523, 4.638)	2.170 (1.936, 2.404)	0.360 (0.251, 0.469)	0.499 (0.444, 0.554)
Ever pregnancy + most healthy	−0.292 (−0.44, −0.144)	−0.218 (−0.291, −0.145)	−0.015 (−0.03, 0)	−0.078 (−0.095, −0.061)
Ever pregnancy + moderately healthy	0.644 (0.484, 0.804)	0.395 (0.316, 0.474)	0.079 (0.062, 0.095)	0.070 (0.051, 0.088)
Ever pregnancy + least healthy	3.018 (2.544, 3.492)	2.057 (1.535, 2.579)	0.314 (0.266, 0.363)	0.494 (0.372, 0.617)
Model 2^b^				
Never pregnancy + most healthy	Reference	Reference	Reference	Reference
Never pregnancy + moderately healthy	0.993 (0.758, 1.228)	0.707 (0.592, 0.822)	0.101 (0.077, 0.125)	0.167 (0.140, 0.194)
Never pregnancy + least healthy	3.396 (2.341, 4.452)	2.004 (1.772, 2.237)	0.340 (0.232, 0.449)	0.467 (0.412, 0.522)
Ever pregnancy + most healthy	−0.273 (−0.42, −0.125)	−0.200 (−0.273, −0.128)	−0.017 (−0.032, −0.002)	−0.081 (−0.098, −0.064)
Ever pregnancy + moderately healthy	0.616 (0.457, 0.776)	0.370 (0.291, 0.448)	0.072 (0.056, 0.088)	0.060 (0.042, 0.078)
Ever pregnancy + least healthy	2.834 (2.360, 3.308)	1.891 (1.372, 2.409)	0.291 (0.243, 0.340)	0.467 (0.345, 0.589)

Data are regression coefficients with their corresponding 95% confidence intervals.

^a^Model 1 was adjusted for chronological age, race and ethnicity, educational level, BMI, and Townsend deprivation index.

^b^Model 2 was further adjusted for Charlson Comorbidity Index based on model 1.

### Causal mediation analyses

**Figure 3:**
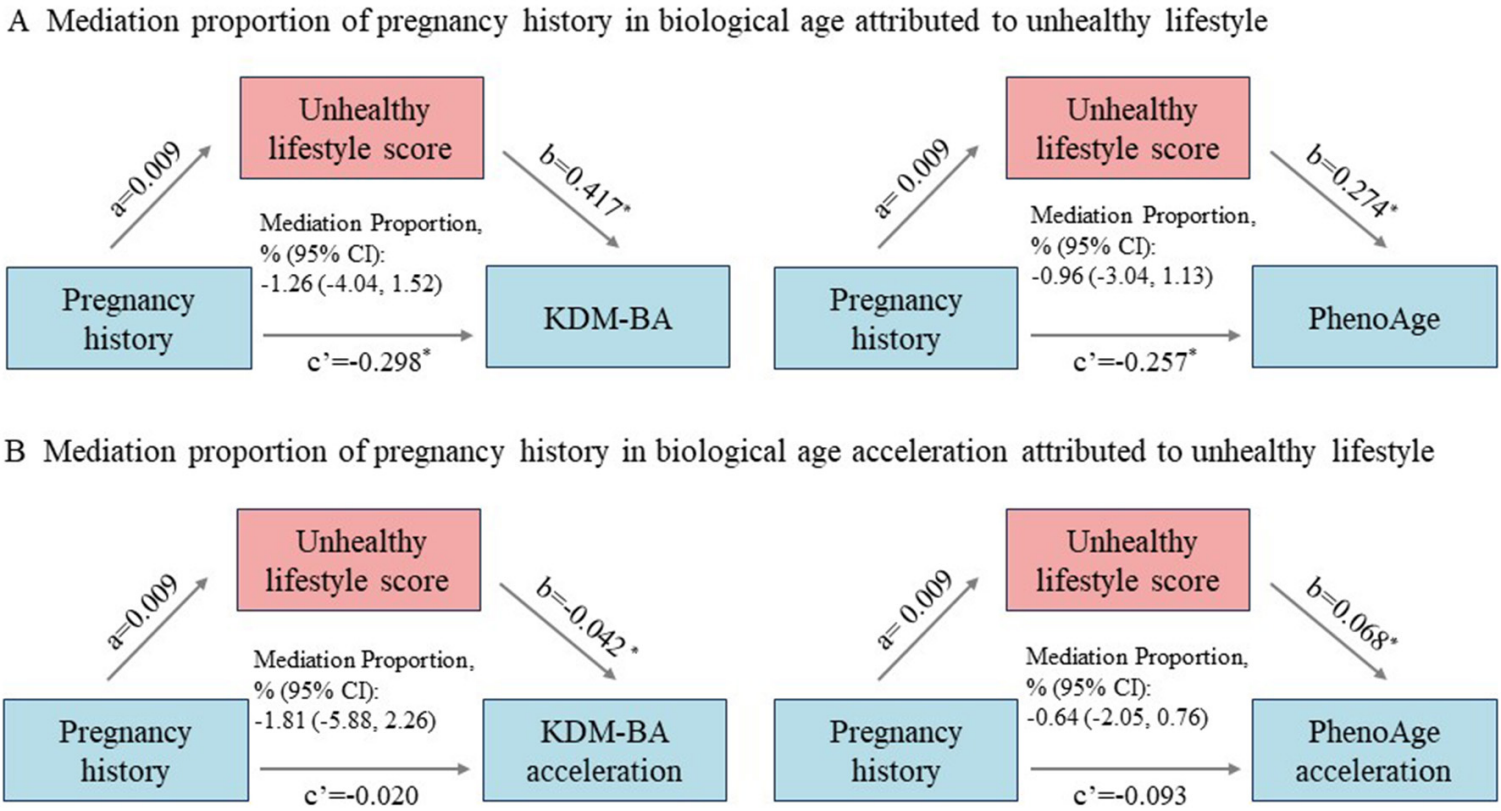
Mediation effects of unhealthy lifestyle on the association of pregnancy history with biological age **(A)** and biological age acceleration **(B)**. The standardized regression coefficients were retrieved from multivariate models with adjustment for covariates, including chronological age, race and ethnicity, educational level, BMI, Townsend deprivation index, Charlson Comorbidity Index, and unhealthy lifestyle score. *P < 0.05.

The lifestyle score did not mediate the association of pregnancy history with BA and BA acceleration (Figure 3). For example, for KDM BA, a moderate total effect [β = −0.294 (95% CI, −0.415 to −0.173)] and direct effect [β = −0.298 (95% CI, −0.419 to −0.177)] were observed, while the indirect effect was non-significant [β = −0.002 (95% CI, −0.003 to 0.008)], with a mediation proportion of −1.26% (95% CI, −4.04 to 1.52). Similarly, for KDM BA acceleration, a smaller total effect [β = −0.030 (95% CI, −0.043 to −0.018)] and direct effect [β = −0.031 (95% CI, −0.043 to −0.018)] were found, with a non-significant indirect effect [β = −0.0003 (95% CI, −0.0004 to 0.001)], yielding a mediation proportion of −1.81% (95% CI, −5.88 to 2.26).

### Sensitive analyses

A series of additional analyses were conducted to evaluate the robustness of the findings. First, stratified analyses showed significant interactions between chronological age and pregnancy history on BA and BA acceleration (Supplementary Table S4). For example, the association between pregnancy history and KDM BA were more pronounced in women aged 39–59 years [β = −0.381 (95% CI, −0.524 to −0.238)] than in those aged 60–71 years [β = −0.134 (95% CI, −0.356 to −0.087); *P* for interaction < 0.001]. Second, when analyses were repeated without adjusting for chronological age, the results changed substantially, as women who with a history of pregnancy had an increase in KDM BA (β = 1.902, 95% CI, 1.738 to 2.066) and PhenoAge (β = 1.870, 95% CI, 1.748 to 1.992) (Supplementary Table S5). Third, analyses conducted using a complete-case sample yielded similar results (Supplementary Table S6). Fourth, women with a higher number of live births or miscarriages had a decrease in BA or BA acceleration, whereas those with more stillbirths had an increase in BA and BA acceleration (Supplementary Table S7). Fifth, participants excluded due to missing data for clinical biomarkers were more likely to be chronologically older, have a higher educational level, a higher TDI, and to be obese, and less likely to be White, compared with the total population (Supplementary Table S8). Finally, additional adjustment for gestational diabetes mellitus, hypertensive disorders of pregnancy, and hormone replacement therapy use resulted in some changes in the magnitude of the associations, while the direction and statistical significance remained consistent (Supplementary Table S9).

## Discussion

In this large UK national cohort of 137,218 female participants, we found that women with a history of pregnancy had a lower BA and BA acceleration than those who had never been pregnant, even after adjusting for sociodemographic, clinical, and lifestyle factors. In the joint analyses, higher BA and BA acceleration were observed among women with unhealthier lifestyle behaviors, regardless of pregnancy history. Nevertheless, no significant interaction and mediation was found between pregnancy history and unhealthy lifestyle score on BA and BA acceleration.

### Comparison with previous studies

Prior prospective and retrospective studies have yielded conflicting results on the relationship between pregnancy and clinical biomarker-based BA (7, 10–12, 14). A cohort study of Swedish pregnant women reported a progressive increase in PhenoAge acceleration from pre-pregnancy to a few days postpartum (11, 12). However, a cross-sectional study of young Filipino women found no significant relationship between gravidity and PhenoAge acceleration (7). Additionally, a study of American women observed no relationship between parity and KDM-BA acceleration in premenopausal women but found an association in postmenopausal women (14). Meanwhile, several studies have suggested that adherence to more health-promoting factors was associated with slower KDM BA and PhenoAge acceleration (25, 26). Our findings suggested that pregnancy was negatively associated with BA and BA acceleration, even after adjusting for sociodemographic, clinical, and lifestyle factors. In addition, a higher unhealthy lifestyle score or engagement in most individual unhealthy lifestyle behaviors was associated with increased BA and BA acceleration. However, no significant interaction or mediation effect was observed between pregnancy history and unhealthy lifestyle score on BA and BA acceleration. These null findings should be interpreted descriptively rather than as evidence against potential biological interplay. Several factors may explain these inconsistent findings. Differences in study design, particularly longitudinal vs. cross-sectional approaches, may capture distinct phases of biological aging. In addition, variation in age, reproductive stage, and parity across study populations may influence observed associations. Finally, the use of different biological age measures and biomarker sets may further contribute to heterogeneity across studies.

### Potential mechanisms

The observed associations between pregnancy history and lower BA may reflect hormonal, immunological, and metabolic adaptations induced by pregnancy; however, given the cross-sectional design, these mechanistic interpretations were descriptive and should be considered hypothesis-generating. Pregnancy triggers significant hormonal shifts, notably increased estrogen and progesterone levels, which exert protective effects on cardiovascular, metabolic, and immune systems (27). Estrogen enhances endothelial function, reduces oxidative stress, and promotes vasodilation (28), while also providing neuroprotection against cognitive decline (29). Progesterone contributes by modulating immune responses and reducing systemic inflammation, a key driver of biological aging (30). Additionally, long-term immune adaptations, including enhanced regulatory T-cell activity and cytokine modulation, may lower chronic inflammation and decelerate biological aging (31). Pregnancy might induce pronounced metabolic adaptations. Although transient insulin resistance occurs during late gestation, evidence indicates that improvements in insulin sensitivity, lipid metabolism, and energy homeostasis may persist postpartum, potentially modulating subsequent biological aging (32). Pregnancy-related epigenetic modifications in immune cells could further enhance immune resilience, influencing the aging process (33). Another plausible mechanism is fetal microchimerism, where fetal cells persist in maternal circulation and various tissues for decades postpartum (34). Studies suggested these fetal cells contribute to maternal tissue repair and regeneration, potentially mitigating age-related deterioration (35). Moreover, pregnancy-induced metabolic adaptations, such as improved insulin sensitivity, lipid metabolism, and energy homeostasis (36, 37), may further slow biological aging long-term metabolic disease risk. Further research is warranted to elucidate the biological pathways linking pregnancy to BA modulation.

### Strengths and limitations

The large sample size of a well-characterized population increased statistical power to identify interactions through multiplicative interaction terms in regression models and stratified analyses. Additionally, the objective assessment of BA using clinical biomarkers, combined with comprehensive adjustments for potential confounding factors, enhanced the validity of our findings. Beyond its large sample size and use of multiple biological aging metrics, a key strength of this study is its life-course–oriented analytical framework. By jointly examining pregnancy history and lifestyle behaviors, we move beyond isolated risk factor analyses to explore how distinct life stages contribute to biological aging. This study has several limitations. First, the pregnancy history and lifestyle behaviors were self-reported, which may lead to some misclassification. Although these self-reported data used in our study have not been validated directly, previous studies have suggested that such data is highly reliable (38, 39). Second, the cross-sectional design limited causal inference because biological age was assessed only at baseline. Given the relatively older age of the cohort, pregnancy history likely reflects long-term reproductive exposure rather than recent physiological changes, and the biomarkers used primarily capture chronic biological processes. Mediation analyses were exploratory, and the temporal ordering between pregnancy, lifestyle factors, and biological aging was uncertain; these results should therefore be interpreted cautiously. Future longitudinal studies with detailed reproductive timing and repeated biomarker assessments are needed to clarify temporal relationships and life-course effects. Third, the study population was volunteer-based and not fully representative of the UK population, which may limit the generalization of the results to other racial or ethnic groups. Fourth, the lack of information on time since pregnancy restricted our ability to assess time-dependent associations with biological aging. Fifth, despite the large sample size, power to detect multiplicative interactions between pregnancy history and unhealthy lifestyle may be limited. Thus, the absence of significant interactions could reflect either a true lack of effect or undetected smaller effects, warranting confirmation in larger studies or with alternative analytic approaches. Sixth, although we used two well-established biological aging measures based on clinical biochemistry biomarkers, other aging measures, such as those derived from proteomic or metabolomic signatures, were not assessed. Future research incorporating these measures may provide further understanding of the complex relationship between pregnancy and aging. In addition, unmeasured psychosocial and environmental factors may contribute to residual confounding and warrant cautious interpretation. Although BA was not a direct clinical outcome, it represented a validated integrative biomarker that captures multisystem physiological aging and was associated with cardiometabolic disease, functional decline, and mortality (40). BA acceleration might therefore serve as an early indicator of long-term disease risk and healthspan. Thereforce, these findings supported a life-course approach that integrated reproductive health and lifestyle modification to promote healthy aging and might contribute to efforts to reduce health inequalities among women.

## Conclusions

With growing recognition of the role of biological aging in health outcomes, optimizing modifiable factors influencing BA may serve as a valuable public health strategy. Our study provides evidence that women with a history of pregnancy had lower BA and BA acceleration, while those with an unhealthier lifestyle had higher BA and BA acceleration, regardless of pregnancy history. These findings highlight the importance of considering these factors in aging-related research and public health interventions. Given the complex interplay between pregnancy, lifestyle, and biological aging, longitudinal studies are needed to further clarify these relationships.

## Data Availability

The datasets presented in this study can be found in online repositories. The names of the repository/repositories and accession number(s) can be found below: https://www.ukbiobank.ac.uk/.
