# Supplementary material for: Association of pregnancy history and unhealthy lifestyle with biological age acceleration: a large cross-sectional study
Source: Front Public Health. 2026 Mar 17;14:1761874. doi: 10.3389/fpubh.2026.1761874 (PMC13036103; doi:10.3389/fpubh.2026.1761874)
Supplement: Supplementary file 1 [file Data_Sheet_1.docx]

**Association of pregnancy history and unhealthy lifestyle with biological age acceleration: a large cross-sectional study**

**Table S1. Variables used to create lifestyle score and scoring system for UK Biobank**

| **Lifestyle factor** | Questionnaire | Not at risk | At risk |
| --- | --- | --- | --- |
| **Cigarette smoking** | Do you smoke tobacco now? and "In the past, how often have you smoked tobacco?" | past or never smoker | current |
| **Alcohol consumption** | “About how often do you drink alcohol?” | ≤ 4 times week | Daily or almost daily |
| **Physical activity** | IPAQ short form2– total time walking or moderate and vigorous-intensity PA in previous week | ≥150 min/week moderate or ≥ 75 min/week vigorous PA | <150 min/week moderate or < 75 min/week vigorous PA |
| **Television viewing /sedentary time** | In a typical day, how many hours do you spend watching TV? | < 4 h/day | ≥ 4 h/day |
| **Sleep duration** | About how many hours sleep do you get in every 24 hours? | >7 or <9h/day | <7 or >9h/day |
| **Dietary characteristics** | Individual dietary components contributed directly to lifestyle score. |  |  |
| **Fruit and vegetable intake** | About how many of …. would you eat per day? Separate questions for pieces of fresh and dried fruit, tablespoons of salad or cooked/raw vegetables. Combined and converted to g/day (1 portion = 80 g) | ≥ 400 g/day | <400 g/ day |
|  |  |  |  |
| **Oily fish intake** | How often do you eat oily fish? (e.g. sardines, salmon, mackerel, herring) | ≥1 portion/week | <1 portion/week |
| **Red meat intake** | How often do you eat…? Separate questions for Beef / lamb or mutton / pork (excluding processed meats such as ham or bacon). Red meat included due to clear link between red meat and mortality. | ≤3 portion/week | >3 portion/week |
| **Processed meat intake** | How often do you eat processed meats (such as bacon, ham, sausages, meat pies, kebabs, burgers, chicken nuggets)? | ≤1 portion/week | >1 portion/week |

**Table S2. Components of biological ages across pregnancy history**

| **Components of biological ages** | **All  (N=137218)** | **Never pregnancy (n=21543)** | **Ever pregnancy (n=115675)** |
| --- | --- | --- | --- |
| FEV1 (L)^*^ | 2.37±0.53 | 2.42±0.54 | 2.36±0.53 |
| SBP (mm Hg)^*^ | 134.03±18.81 | 132.38±18.27 | 134.33±18.89 |
| Total Cholesterol (mg/dL)^*^ | 227.02±42.67 | 224.93±42.16 | 227.41±42.76 |
| Glycated hemoglobin (%)^*^ | 5.39±0.47 | 5.36±0.49 | 5.39±0.47 |
| Blood urea nitrogen (mg/dL)^*^ | 14.53±3.5 | 14.12±3.52 | 14.6±3.49 |
| Lymphocyte (%)^#^ | 29.88±7.18 | 29.27±7.16 | 29.99±7.18 |
| Mean cell volume (fL)^#^ | 82.98±5.18 | 83.43±5.28 | 82.89±5.16 |
| Serum glucose (mg/dL)^#^ | 90.41±16.11 | 90.33±16.69 | 90.43±16.01 |
| Red cell distribution width (%)^#^ | 13.48±0.95 | 13.46±0.95 | 13.48±0.95 |
| White blood cell count (1000 cells/uL)^#^ | 6.77±1.71 | 6.82±1.77 | 6.76±1.7 |
| Albumin (g/dL)^*#^ | 4.5±0.26 | 4.5±0.26 | 4.51±0.25 |
| Creatinine (mg/dL)^*#^ | 0.72±0.12 | 0.72±0.12 | 0.72±0.12 |
| C-reactive protein (mg/dL)^*#^ | 0.24±0.36 | 0.24±0.37 | 0.24±0.36 |
| Alkaline phosphatase (U/L)^*#^ | 82.78±23.77 | 80.75±23.88 | 83.16±23.73 |

^*^Used to calculate biological age by using the Klemera-Doubal method (KDM).

^#^Used to calculate PhenoAge algorithm.

**Table S3. Associations of lifestyle with biological age and biological age acceleration by individual lifestyle factors^a^**

|  | KDM BA | PhenoAge | KDM BA acceleration | PhenoAge acceleration |
| --- | --- | --- | --- | --- |
| **Cigarette smoking** |  |  |  |  |
| Not at risk | Ref | Ref | Ref | Ref |
| At risk | 1.314 (1.152, 1.476) | 1.77 (1.691, 1.849) | 0.125 (0.108, 0.141) | 0.444 (0.426, 0.463) |
| **Alcohol consumption** |  |  |  |  |
| Not at risk | Ref | Ref | Ref | Ref |
| At risk | -0.226 (-0.342, -0.11) | -0.195 (-0.252, -0.139) | -0.017 (-0.028, -0.005) | -0.066 (-0.079, -0.053) |
| **Physical activity** |  |  |  |  |
| Not at risk | Ref | Ref | Ref | Ref |
| At risk | 0.079 (-0.009, 0.167) | 0.034 (-0.009, 0.077) | 0.005 (-0.004, 0.014) | 0.019 (0.009, 0.029) |
| **Television viewing/**  **sedentary time** |  |  |  |  |
| Not at risk | Ref | Ref | Ref | Ref |
| At risk | 0.959 (0.855, 1.063) | 0.34 (0.289, 0.39) | 0.113 (0.103, 0.124) | 0.034 (0.023, 0.046) |
| **Sleep duration** |  |  |  |  |
| Not at risk | Ref | Ref | Ref | Ref |
| At risk | 0.193 (0.091, 0.295) | 0.068 (0.018, 0.117) | 0.023 (0.013, 0.034) | 0.005 (-0.006, 0.017) |
| **Fruit and vegetable intake** |  |  |  |  |
| Not at risk | Ref | Ref | Ref | Ref |
| At risk | 1.156 (0.995, 1.318) | 0.532 (0.453, 0.611) | 0.11 (0.094, 0.127) | 0.15 (0.132, 0.169) |
| **Oily fish intake** |  |  |  |  |
| Not at risk | Ref | Ref | Ref | Ref |
| At risk | -0.209 (-0.299, -0.119) | 0.213 (0.169, 0.258) | -0.031 (-0.04, -0.022) | 0.079 (0.069, 0.09) |
| **Red meat c intake** |  |  |  |  |
| Not at risk | Ref | Ref | Ref | Ref |
| At risk | 0.739 (0.606, 0.872) | 0.057 (-0.008, 0.122) | 0.077 (0.064, 0.091) | 0.009 (-0.006, 0.024) |
| **Processed meat intake** |  |  |  |  |
| Not at risk | Ref | Ref | Ref | Ref |
| At risk | 0.739 (0.629, 0.849) | 0.331 (0.277, 0.385) | 0.072 (0.061, 0.084) | 0.088 (0.075, 0.1) |

Data are regression coefficients with their corresponding 95% confidence intervals.

^a^ Model was adjusted for race and ethnicity, educational level, BMI, and Townsend deprivation index, Charlson Comorbidity Index, and lifestyle factors (smoking status, alcohol intake, physical activity, TV viewing /sedentary time, Sleep time, fruit and vegetable intake, oily fish intake, red meat intake, and processed meat intake).

**Table S4. Associations between pregnancy history and biological age and biological age acceleration by chronological age^a^**

| **Pregnancy history**  **(Ever vs, never)** | KDM BA | PhenoAge | KDM BA acceleration | PhenoAge acceleration |
| --- | --- | --- | --- | --- |
| 39–59 | -0.381 (-0.524, -0.238) | -0.241 (-0.312, -0.169) | -0.027 (-0.042, -0.013) | -0.082 (-0.099, -0.065) |
| 60–71 | -0.134 (-0.356, -0.087) | -0.227 (-0.332, -0.122) | -0.017 (-0.039, 0.006) | -0.051 (-0.075, -0.026) |
| *P* for interaction | <0.001 | <0.001 | <0.001 | <0.001 |

Data are regression coefficients with their corresponding 95% confidence intervals.

^a^ Model was adjusted for chronological age, race and ethnicity, educational level, BMI, and Townsend deprivation index, Charlson Comorbidity Index, and unhealthy lifestyle score.

**Table S5. Associations of pregnancy history with biological age, without adjusting for chronological age**

|  | KDM BA | PhenoAge |
| --- | --- | --- |
| **Model 1^a^** |  |  |
| Never pregnancy | Reference | Reference |
| Ever pregnancy | 1.999 (1.832, 2.166) | 1.961 (1.836, 2.087) |
| **Model 2^b^** |  |  |
| Never pregnancy | Reference | Reference |
| Ever pregnancy | 1.901 (1.737, 2.065) | 1.870 (1.748,1.992) |
| **Model 3^c^** |  |  |
| Never pregnancy | Reference | Reference |
| Ever pregnancy | 1.902 (1.738, 2.066) | 1.870 (1.748, 1.992) |

Data are regression coefficients with their corresponding 95% confidence intervals.

^a^ Model 1 was adjusted for race and ethnicity, educational level, BMI, and Townsend deprivation index.

^b^ Model 2 was further adjusted for Charlson Comorbidity Index based on model 1.

^c^ Model 3 was further adjusted for unhealthy lifestyle score based on model 2.

**Table S6. Associations of pregnancy history with biological age and biological age acceleration, with mediation proportion of pregnancy history in biological age acceleration attributed to unhealthy lifestyle in a complete-case sample (N=136 058)**

|  | KDM BA | PhenoAge | KDM BA acceleration | PhenoAge acceleration |
| --- | --- | --- | --- | --- |
| **Model 1^a^** |  |  |  |  |
| Never pregnancy | Reference | Reference | Reference | Reference |
| Ever pregnancy | -0.319 (-0.441, -0.197) | -0.250 (-0.310, -0.189) | -0.019 (-0.031, -0.006) | -0.085 (-0.099, -0.071) |
| **Model 2^b^** |  |  |  |  |
| Never pregnancy | Reference | Reference | Reference | Reference |
| Ever pregnancy | -0.308 (-0.430, -0.187) | -0.240 (-0.300, -0.180) | -0.021 (-0.034, -0.009) | -0.089 (-0.103, -0.075) |
| **Model 3^c^** |  |  |  |  |
| Never pregnancy | Reference | Reference | Reference | Reference |
| Ever pregnancy | -0.313 (-0.435, -0.192) | -0.243 (-0.303, -0.184) | -0.021 (-0.034, -0.009) | -0.089 (-0.103, -0.075) |

Data are regression coefficients with their corresponding 95% confidence intervals.

^a^ Model 1 was adjusted for chronological age, race and ethnicity, educational level, BMI, and Townsend deprivation index.

^b^ Model 2 was further adjusted for Charlson Comorbidity Index based on model 1.

^c^ Model 3 was further adjusted for unhealthy lifestyle score based on model 2.

**Table S7. Associations of reproductive factors with biological age and biological age acceleration^a^**

|  | KDM-BA | PhenoAge | KDM-BA acceleration | PhenoAge acceleration |
| --- | --- | --- | --- | --- |
| Number of live births | -0.162 (-0.200, -0.125) | -0.045 (-0.063, -0.027) | -0.011 (-0.015, -0.007) | -0.026 (-0.030, -0.020) |
| Number of stillbirth | 0.578 (0.350, 0.805) | 0.252 (0.139, 0.364) | 0.068 (0.044, 0.091) | 0.029 (0.002, 0.056) |
| Number of miscarriages | -0.216 (-0.264, -0.168) | -0.005 (-0.029, 0.018) | -0.025 (-0.030, -0.020) | 0.007 (-0.002, 0.013) |

Data are regression coefficients with their corresponding 95% confidence intervals.

^a^ Model was adjusted for chronological age, race and ethnicity, educational level, BMI, Townsend deprivation index, Charlson Comorbidity Index, and unhealthy lifestyle score.

**Table S8. Baseline characteristics of participants excluded because of missing data on clinical biomarkers (used to calculate phenotypic age acceleration) and the total population who participated in the baseline survey**

| **Characteristic** | **Total population (N=137218)** | **Participants excluded (n=59594)** | ***P*** |
| --- | --- | --- | --- |
| **Chronological age** | 55.73±8.01 | 56.15±8.05 | <0.001 |
| **Race and ethnicity** |  |  | <0.001 |
| White | 131175 (95.6) | 55894 (93.8) |  |
| Non-Whitea | 5784 (4.2) | 3563 (6.0) |  |
| Unknown | 259 (0.2) | 137 (0.2) |  |
| **Educational level^b^** |  |  | <0.001 |
| High | 34789 (25.4) | 15490 (26.0) |  |
| Intermediate | 84276 (61.4) | 35389 (59.4) |  |
| Low | 17434 (12.7) | 8363 (14.0) |  |
| Unknown | 719 (0.5) | 352 (0.6) |  |
| **Townsend deprivation index quintile^c^** | 0.85±1.54 | 1.04±1.7 | <0.001 |
| **BMI at recruitment (kg/m^2^)** |  |  | <0.001 |
| <18.5 | 1017 (0.7) | 491 (0.8) |  |
| 18.5–24.9 | 56996 (41.5) | 23208 (38.9) |  |
| 25–29.9 | 50265 (36.6) | 21428 (36.0) |  |
| ≥30 | 28747 (20.9) | 13867 (23.3) |  |
| Unknown | 193 (0.1) | 600 (1.0) |  |

^a^ Other includes any races or ethnicities not specifically categorized.

^b^ Educational attainment was categorized into three levels: high, defined as holding a college or university degree; intermediate, which includes advanced (A/AS) levels or equivalent, ordinary (O) level, general certificate of secondary education, or equivalent qualifications—corresponding to grades 6–12 in the US education system, where O-level is equivalent to middle school (grades 6–8) and A/AS-level corresponds to high school (grades 9–12); and low, referring to individuals without any of the aforementioned qualifications.

^c^ The Townsend Deprivation Index is a measure of socioeconomic status, where 0 represents the mean value for an area, positive values indicate lower socioeconomic status, and negative values indicate higher socioeconomic status.

^d^ Body mass index was calculated as body weight in kilogram divided by squared of height in meter.

**Table S9. Associations of pregnancy history with biological age and biological age acceleration, with adjustment for gestational diabetes** **mellitus, hypertensive disorders of pregnancy, and hormone replacement therapy**

|  | KDM BA | PhenoAge | KDM BA acceleration | PhenoAge acceleration |
| --- | --- | --- | --- | --- |
| Never pregnancy | Reference | Reference | Reference | Reference |
| Ever pregnancy | -0.026 (-0.038, -0.013) | -0.080 (-0.093, -0.066) | -0.351 (-0.472, -0.230) | -0.237 (-0.297, -0.178) |

Data are regression coefficients with their corresponding 95% confidence intervals.

Adjusted for chronological age, race and ethnicity, educational level, BMI, Townsend deprivation index, Charlson Comorbidity Index, lifestyle score, and gestational diabetes mellitus, hypertensive disorders of pregnancy, and hormone replacement therapy.
